# Quantification of gastric emptying caused by impaired coordination of pyloric closure with antral contraction: a simulation study

**DOI:** 10.1098/rsif.2019.0266

**Published:** 2019-08-07

**Authors:** Shunichi Ishida, Taimei Miyagawa, Gregory O'Grady, Leo K. Cheng, Yohsuke Imai

**Affiliations:** 1 Graduate School of Engineering, Kobe University, 1-1 Rokkodai, Nada, Kobe 657-8501, Japan; 2 Graduate School of Science and Technology, Hirosaki University, Hirosaki, Japan; 3 Auckland Bioengineering Institute, University of Auckland, Auckland, New Zealand; 4 Riddet Institute, Palmerston North, New Zealand

**Keywords:** gastric motor functions, rapid gastric emptying, bile reflux, computational fluid dynamics

## Abstract

Proper coordination of gastric motor functions is required for healthy gastric emptying. However, pyloric function may be impaired by functional disorders or surgical procedures. Here, we show how coordination between pyloric closure and antral contraction affects the emptying of liquid contents. We numerically simulated fluid dynamics using an anatomically realistic gastrointestinal geometry. Peristaltic contractions in the proximal stomach resulted in gastric emptying at a rate of 3–8 ml min^−1^. When the pylorus was unable to close, the emptying rate increased to 10–30 ml min^−1^, and instantaneous retrograde flow from the duodenum to the antrum occurred during antral relaxation. Rapid emptying occurred if the pylorus began to open during the terminal antral contraction, and the emptying rate was negative if the pylorus only opened during the antral relaxation phase. Our results showed that impaired coordination between antral contraction and pyloric closure can result in delayed gastric emptying, rapid gastric emptying and bile reflux.

## 1. Introduction

Gastric mixing and emptying are modulated by gastric motor functions, including the peristaltic and tonic contractions of the stomach and opening and closure of the pylorus. A number of studies have been conducted to investigate the relationship between gastroduodenal motility and the emptying of gastric contents [1–6]. Closure of the pylorus is coordinated with antral contractions [7,8]. When a peristaltic contraction reaches the terminal segment of the antrum, the terminal antrum contracts near simultaneously, called terminal antral contraction, and the pylorus closes near the onset of the terminal antral contraction. Gastric contents in the terminal antrum are then forced back into the proximal antrum. The antrum then relaxes, and the pylorus begins to open. Flow from the antrum to the duodenum is driven by a pressure gradient across the pylorus, and flow through the pylorus has been observed even without contraction of the distal antrum [9–12]. A question in gastrointestinal physiology is to what extent gastric emptying is produced by peristaltic contractions of the antrum, tonic contractions of the fundus or the hydrostatic pressure difference between the antrum and the duodenum. These factors may appear simultaneously *in vivo*, and it is experimentally difficult to isolate and define the relative contribution of each factor to gastric emptying.

Pyloric opening and closure are not always coordinated with antral contractions. Impaired coordination may alter flow patterns, potentially promoting or impeding gastric emptying, or contributing to retrograde flow from the duodenum to the antrum and bile reflux. Surgical procedures such as pyloromyotomy and pyloroplasty also impair pyloric function. Hence, it is important to understand what happens when pyloric function is diminished or compromised. Advanced medical imaging techniques [13,14] have enabled us to visualize the distribution of gastric contents in the stomach [15,16] and the shape and velocity of peristaltic contractions of the antrum [17–19]. However, information on gastric flow available from medical imaging is limited; hence, the relationship between gastric motility and gastric mixing and emptying has not been fully clarified.

Computational modelling and simulations of gastric flow have been performed, enabling integrative and quantitative *in silico* studies that cannot be easily performed experimentally [19–23]. We previously developed a computational fluid dynamics model of gastric mixing, using an anatomically realistic geometry of the stomach [24]. We have also applied this model to successfully quantify gastric mixing produced by antral contractions [25,26]. Here, we extend this model to quantify the relationship between gastric motility and gastric mixing and emptying, particularly focusing on impaired coordination of the pyloric closure with antral contractions.

## 2. Methods

### 2.1. Gastroduodenal geometry and antral contraction

An anatomically realistic gastroduodenal model based on data from the Visible Human Project [27,28] was used. The total volume of the stomach was approximately 650 ml, and the average diameter of the antrum was approximately *D* = 50 mm. The diameter of the pylorus was 9 mm when open. Here, we concentrated on gastric mixing and emptying produced by antral contractions, and we omitted tonic contractions of the stomach.

Each antral contraction consisted of three phases: peristaltic contraction, terminal antral contraction and antral relaxation. A peristaltic contraction initiated at the mid-corpus, and travelled towards the terminal antrum. Parameters of the contraction wave were identified based on published magnetic resonance imaging (MRI) studies [19]. For healthy subjects, the propagation velocity of the contraction wave was *V* = 2.5 mm s^−1^, and the cycle of the contraction was *T* = 20 s. The contraction width was 18 mm, and the contraction ratio (degree of occlusion) increased as the wave progressed towards the distal antrum.

**Figure 1.**
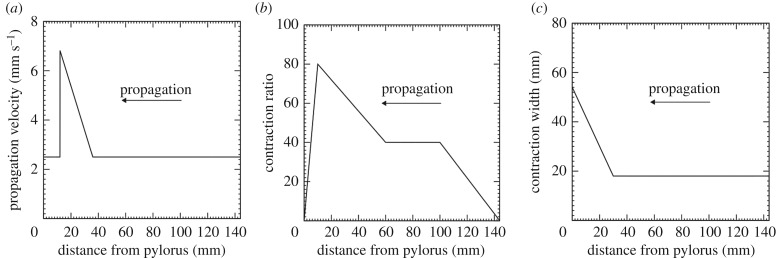
Parameters of contraction waves. (*a*) Propagation velocity, (*b*) contraction ratio and (*c*) contraction width.

The terminal antral contraction refers to a segmental and near-simultaneous contraction of the terminal region of the antrum [7]. When peristaltic contractions reach the terminal antrum, terminal antral contraction occurs, followed by antral relaxation. Because the propagation velocity increases at the terminal antrum [25,29,30], the terminal antral contraction was modelled as an acceleration of the propagation velocity and an increase in the contraction width at the terminal antrum. When peristaltic contractions reached 30 mm from the pylorus, the contraction width linearly increased from 18 to 54 mm. The propagation velocity, the contraction ratio and the contraction width are shown in figure 1.

### 2.2. Impaired coordination of pyloric closure with antral contraction

Carlson *et al*. [7] investigated the coordination between pyloric closure and the terminal antral contraction. The pyloric canal was open during 28% of the total period of observation, and pyloric closure occurred at nearly the same time as the onset of the terminal antral contraction. To model impaired coordination of pyloric closure with antral contraction, we defined two parameters: the duration of pyloric closure, *T*_C_, and the delay of pyloric closure from the onset of terminal antral contraction, *T*_D_. For example, the duration *T*_C_/*T* = 0 corresponded to an impaired function of the pylorus, where the pylorus was unable to close or remained permanently open as a result of surgical procedures. In our control model, the duration of pyloric closure was set to *T*_C_/*T* = 2/3, and the pylorus began to close at *t*/*T* = 0.6, which has no delay from the onset of the terminal antral contraction, *T*_D_/*T* = 0. In impaired coordination models, we varied these two parameters (0 ≤ *T*_C_/*T* ≤ 1 and 0 ≤ *T*_D_/*T* ≤ 1).

### 2.3. Fluid dynamics and numerical methods

An incompressible Newtonian liquid was considered for gastric contents. At the initial state, 80% of the stomach and the whole duodenum were filled with liquid, while the rest of the stomach and the oesophagus were filled with air. While the density of the liquid was constant *ρ* = 1.0 × 10^3^ kg m^−3^, the viscosity of the liquid was varied to examine the effect of viscosity. Viscous traction of air is negligible, and free-surface flow modelling was applied to this problem. The average vertical position of the air–liquid interface was obtained from the emptied volume of the liquid. Assuming that the air–liquid interface remained flat during the process, an external force was applied if the vertical positions differed from the average vertical position. To ignore the effect of hydrostatic pressure difference between the antrum and the duodenum on gastric emptying, hydrostatic equilibrium was also considered. The position and velocity of the gastrointestinal wall were prescribed, and given by moving boundary conditions. We solved the fluid dynamics using the multiple-relaxation-time lattice Boltzmann method [31], with uniform Cartesian grids of 1.5 mm spacing. To implement the moving and curved wall boundary conditions, a bounce-back scheme [32] was applied. Distribution functions at the air–liquid interface were extrapolated [33]. The computation was accelerated using graphics processing unit computing [34]. The emptying rate was computed by the outflow flux at the pylorus. Mixing efficiency was defined as the spatial average of the second invariants of the strain tensor for the cycle of the antral contraction. For more details, see [25,26].

## 3. Results

### 3.1. Gastric emptying produced by peristaltic contractions of the proximal antrum

**Figure 2.**
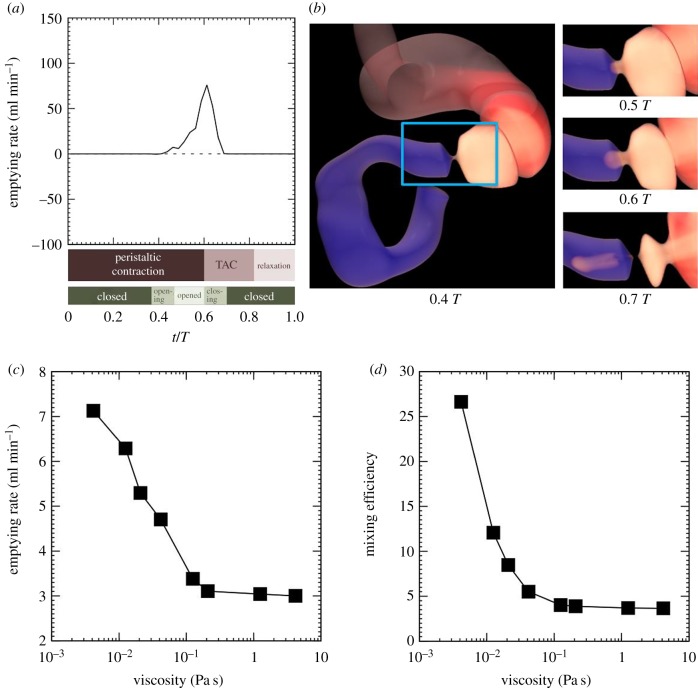
Gastric emptying and mixing for a control case. (*a*) The time history of the instantaneous emptying rate and the time chart of antral contraction and pyloric closure, where *t* is the time and *T* is the period of antral contractions. Content viscosity was 4.2 mPa s. (*b*) Corresponding snapshots where white and red contents were initially located in the stomach and blue contents were initially located in the duodenum. (*c*) Time-averaged emptying rate and (*d*) mixing efficiency, as a function of liquid viscosity. (Online version in colour.)

**Figure 3.**
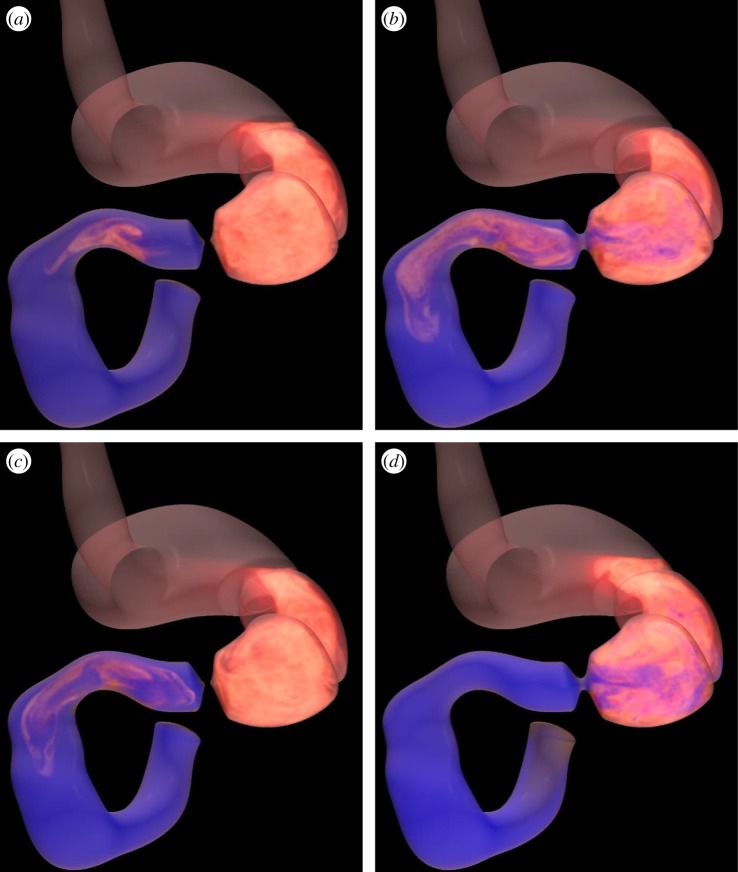
Location of gastric contents after 1 min, where white and red contents were initially located in the stomach and blue contents were initially located in the duodenum. (*a*) Control case (*T*_C_/*T* = 2/3, *T*_D_/*T* = 0). (*b*) The pylorus was unable to close (*T*_C_/*T* = 0, *T*_D_/*T* = 0). (*c*) Impaired coordination (*T*_C_/*T* = 2/3, *T*_D_/*T* = 1/8). (*d*) Impaired coordination (*T*_C_/*T* = 2/3, *T*_D_/*T* = 3/8). (Online version in colour.)

We first examined gastric emptying of a liquid with a low viscosity, *μ* = 4.2 × 10^−3^ Pa s, for a control case, where *T*_C_/*T* = 2/3 and *T*_D_/*T* = 0. Figure 2*a* shows the time history of the instantaneous emptying rate, and the time chart of antral contraction and pyloric closure, where *t*/*T* is the time normalized by the period of antral contraction. A peristaltic contraction reached the proximal antrum at *t*/*T* = 0, and continued to move towards the pylorus (peristaltic contraction). The pylorus began to open at *t*/*T* = 0.4. While the peristaltic contraction still travelled in the proximal antrum, emptying of the liquid contents began (see also snapshots in figures 2*b* and figure 3*a*; electronic supplementary material, video 1). At *t*/*T* = 0.6, the terminal antral contraction began, and the pylorus began to close. The instantaneous emptying rate then sharply decreased. The terminal antral contraction finished at around *t*/*T* = 0.8, and the terminal antrum was relaxed until *t*/*T* = 1.0 (antral relaxation).

To investigate the effect of liquid viscosity on gastric emptying, the viscosity was varied from 4.2 × 10^−3^ to 4.2 Pa s. Examples of liquid foods with a viscosity of *O* (10^−3^) Pa s include water, milk and beer, and those with a viscosity of *O*(1) Pa s include molasses and honey. Figure 2*c* shows the time-averaged emptying rate, as a function of the liquid viscosity. The emptying rate decreased as the content viscosity is increased, but the emptying rate became nearly constant for viscosities higher than *μ* ∼ 0.1 Pa s. The emptying rate ranged from 3 to 8 ml min^−1^ for the control case. We also calculated mixing efficiency and found that the mixing efficiency had similar trends to the emptying rate, as shown in figure 2*d*.

### 3.2. Emptying rate increases but retrograde flow from the duodenum occurs when the pylorus is unable to close

**Figure 4.**
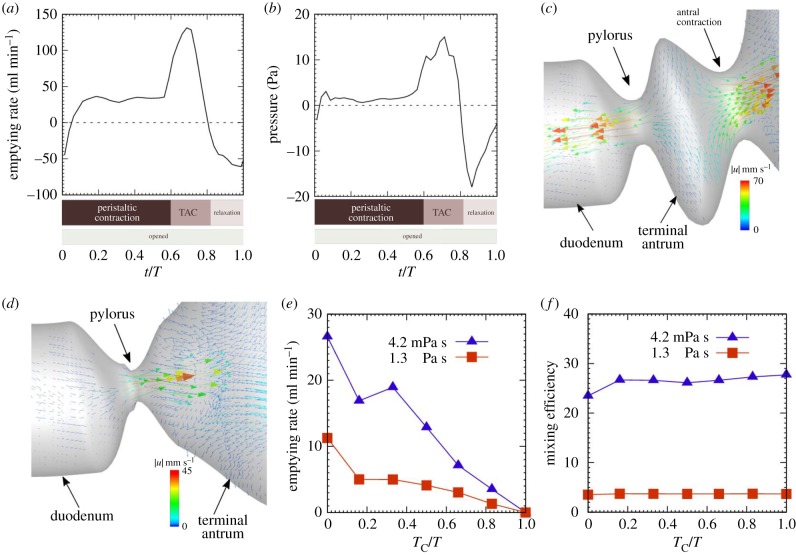
Effects of pyloric closure duration. (*a*) Instantaneous emptying rate and (*b*) pressure difference between the antrum and duodenum for *T*_C_/*T* = 0 (the pylorus is unable to close). (*c*) Velocity vectors during terminal antral contraction and (*d*) those during antral relaxation. (*e*) Time-averaged emptying rate and (*f*) mixing efficiency, as a function of *T*_C_/*T* for two values of content viscosity. (Online version in colour.)

We next investigated the effects of the duration of pyloric closure on gastric mixing and emptying. To check the relationship between the emptying rate and the pressure difference between the antrum and duodenum, we first considered a case where the pylorus was unable to close (i.e. the pylorus remained open; see also figure 3*b*; electronic supplementary material, video 2). We calculated the pressure difference between two locations 5 mm either side of the pylorus (i.e. in the distal antrum and proximal duodenum). The instantaneous emptying rate and the pressure difference are shown for low-viscosity liquid (*μ* = 4.2 × 10^−3^ Pa s) in figure 4*a*,*b*. The pressure difference between the antrum and duodenum promoted a near-constant emptying rate during the peristaltic contraction. The terminal antral contraction increased the pressure in the terminal antrum, and high-velocity flows appeared from there towards the proximal antrum and the duodenum (figure 4*c*). A large pressure difference during the terminal antral contraction resulted in an increase in the instantaneous emptying rate. When the antral relaxation began, however, the pressure difference became negative, i.e. pressure at the duodenum was higher than that at the antrum. Retrograde flow from the duodenum to the antrum then occurred (figure 4*d*), and the instantaneous emptying rate was negative. In the case of low-viscosity contents, negative emptying continued to the beginning of the antral contraction due to inertial effects.

We then varied the duration of pyloric closure from *T*_C_/*T* = 0 (always open) to *T*_C_/*T* = 1 (always closed), while we fixed the timing of the onset of pyloric closure to be the same as the control case. The time-averaged emptying rate is shown in figure 4*e* for two types of viscosities. As expected, the emptying rate tended to decrease when the duration of pyloric closure increased. The emptying rate was maximum at *T*_C_/*T* = 0: 10 ml min^−1^ for high-viscosity contents (*μ* = 1.3 Pa s), and 27 ml min^−1^ for lower viscosity contents (*μ* = 4.2 × 10^−3^ Pa s). The emptying rate for *T*_C_/*T* = 1/6 was slightly lower than *T*_C_/*T* = 1/3 for the low-viscosity liquid. In the case of *T*_C_/*T* = 1/6, pyloric opening began at the end of the terminal antral contraction. The pylorus was fully open during antral relaxation, and the time-averaged emptying rate was decreased by retrograde flow from the duodenum. In contrast with gastric emptying, the duration of pyloric closure had a minor effect on gastric mixing. The mixing efficiency remained nearly constant even when the duration changed from *T*_C_/*T* = 0 to *T*_C_/*T* = 1, as shown in figure 4*f*.

### 3.3. Impaired coordination between pyloric closure and the terminal antral contraction

**Figure 5.**
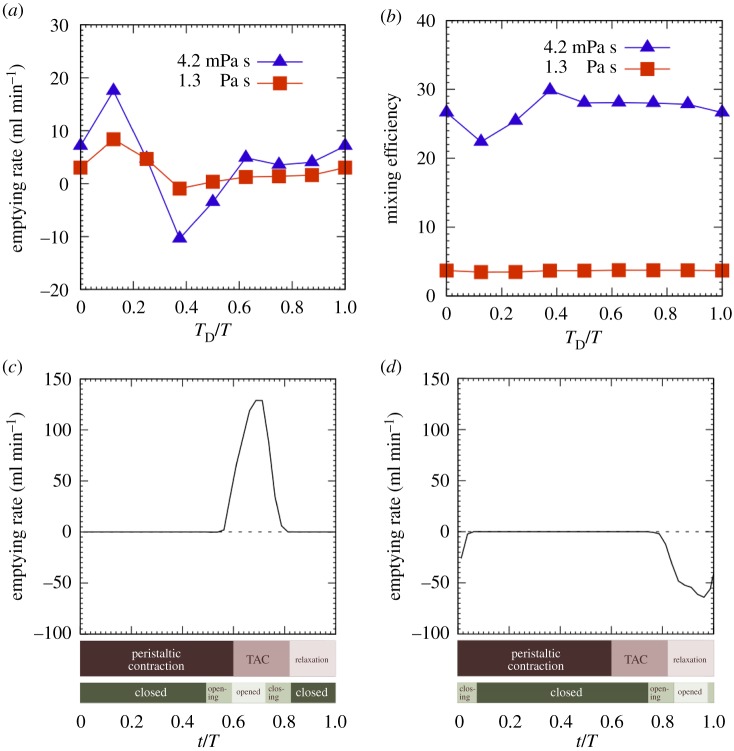
Effects of the delay of pyloric closure. (*a*) Time-averaged emptying rate and (*b*) mixing efficiency, as a function of *T*_D_/*T* for two values of content viscosity. (*c*) Instantaneous emptying rate for *T*_D_/*T* = 1/8, and (*d*) that for *T*_D_/*T* = 3/8. (Online version in colour.)

We then examined impaired coordination of pyloric closure with the antral contraction. We gave a delay, *T*_D_, in the onset of pyloric closure from that of the terminal antral contraction, whereas we fixed the duration of pyloric closure to be *T*_C_/*T* = 2/3 (the same as the control case). The time-averaged emptying rate and the mixing efficiency are shown in figure 5 as a function of the delay of pyloric closure. The delay in pyloric closure drastically altered the time-averaged emptying rate. A delay of *T*_D_/*T* = 1/8 increased the emptying rate two- to threefold for both low- and high-viscosity contents (see also figure 3*c*; electronic supplementary material, video 3). A delay of *T*_D_/*T* = 3/8, however, resulted in a negative value in the emptying rate, in particular for the low-viscosity contents (see also figure 3*d*; electronic supplementary material, video 4). We compare the instantaneous emptying rates between these cases in figure 5*c*,*d*. In the case of *T*_D_/*T* = 1/8, the pylorus was opened during the terminal antral contraction, resulting in rapid emptying. On the other hand, when the delay was *T*_D_/*T* = 3/8, the pylorus was only open during antral relaxation. Flow through the pylorus was always retrograde from the duodenum. The time-averaged emptying rate was thus a negative value. Note that the mixing efficiency was altered for the low-viscosity content by the delay in pyloric closure but only slightly.

To gain a more comprehensive understanding of the effects of impaired coordination, we varied both the duration and delay of pyloric closure, and, hereafter, we present our results in a non-dimensional form. Gastric flow was characterized by two dimensionless numbers in fluid mechanics, the Strouhal number, *St* = *D*/*VT*, and the Reynolds number, *Re* = *ρVD*/*μ*. In this study, the Strouhal number was fixed to *St* = 1, and the Reynolds number depended on the liquid viscosity; for example, *Re* = 0.1 for *μ* = 1.3 Pa s, and *Re* = 30 for *μ* = 4.2 × 10^−3^ Pa s. When the time-averaged emptying rate is denoted by *Q*, the emptying rate can be written as *Q*/*D*^2^*V* in a non-dimensional form.

**Figure 6.**
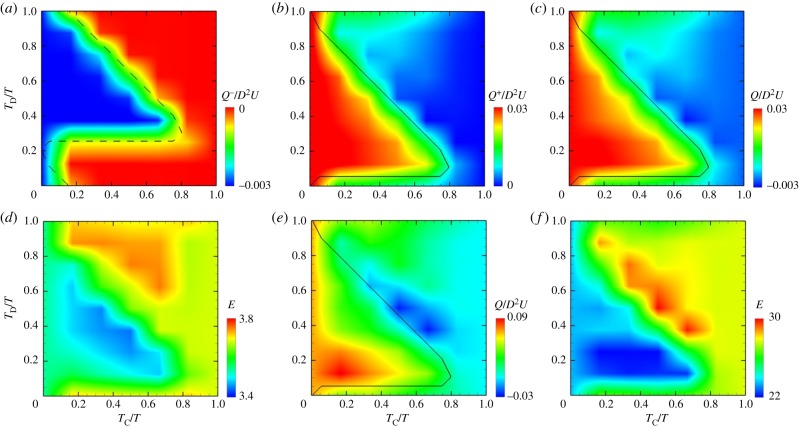
(*a*) Retrograde component, (*b*) anterograde component, (*c*) time-averaged emptying rate and (*d*) mixing efficiency as functions of *T*_C_/*T* and *T*_D_/*T* for *Re* = 0.1. (*e*) Time-averaged emptying rate and (*f*) mixing efficiency for *Re* = 30. (Online version in colour.)

Total gastric emptying is a consequence of anterograde flow from the antrum to the duodenum, and, if present, retrograde flow from the duodenum to the antrum. The retrograde component, *Q*^−^/*D*^2^*V*, is shown for *Re* = 0.1 in figure 6*a* as a function of *T*_C_/*T* and *T*_D_/*T*, where the region on the left of the dashed line indicates cases when the pylorus is open during antral relaxation (longer than half the relaxation period). This region corresponds well to large values in the retrograde component. This result suggests that the pylorus must be closed during antral relaxation to prevent retrograde flow through the pylorus. The anterograde component, *Q* ^+^/*D*^2^*V*, is also shown in figure 6*b*. When the pylorus was open during the terminal antral contraction (region left of the solid line), the anterograde component was a large value, resulting in rapid emptying, as shown in figure 6*c*. The duration of pyloric closure and its coordination with the terminal antral contraction had a minor effect on gastric mixing over the cases examined in this study. The difference between the maximum and minimum values in mixing efficiency was approximately 10% (figure 6*d*). For *Re* = 30, we found the same tendencies in the emptying rate and mixing efficiency as for *Re* = 0.1, with a small difference due to inertia effects (figure 6*e,f*).

### 3.4. Is gastric content first emptied near the pylorus?

**Figure 7.**
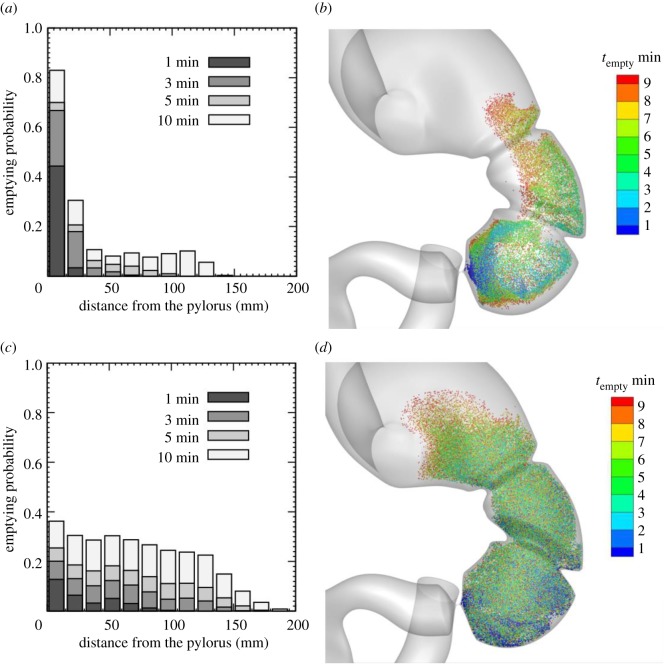
(*a*,*c*) Emptying probability and (*b*,*d*) initial position of tracer particles which have emptied within 10 min for (*a*,*b*) *Re* = 0.1 and (*c*,*d*) *Re* = 30. (Online version in colour.)

Finally, we investigated the region where gastric contents are first emptied. We initially positioned 480 000 tracer particles randomly in the stomach, and simulated the movement of these particles for 10 min. The duration and delay of pyloric closure were set to the control values of *T*_C_/*T* = 2/3 and *T*_D_/*T* = 0. We divided the whole stomach into 13 regions based on the distance from the pylorus, and calculated ‘emptying probability’ as the percentage of emptied particles in each region for a given time. Figure 7*a* shows the emptying probability for content with a high viscosity. More than 60% of the high-viscosity content initially located at the terminal antrum was emptied within 3 min, with approximately 20% remaining in the stomach even after 10 min. Figure 7*b* shows the initial position of the tracer particles which had emptied within 10 min. For high-viscosity contents, the content along the greater curvature emptied in preference to content located along the lesser curvature. However, for low-viscosity contents, such a tendency was diminished. Low-viscosity contents were mixed homogeneously in the antrum and corpus within a few minutes, and, thus, the content was uniformly emptied from these regions, as shown in figure 7*c*,*d*.

## 4. Discussion

In this study, we investigated the mechanism of gastric emptying using an anatomically realistic geometry of the stomach and duodenum. The results showed that peristaltic contractions at the proximal antrum produced gastric emptying with an emptying rate of 3 ml min^−1^ for highly viscous liquids (*μ* > 0.1 Pa s) under hydrostatic equilibrium. The emptying rate was increased when the viscosity was decreased. The emptying rate ranged from 3 to 8 ml min^−1^ for liquid contents (0.001 Pa s < *μ* < 0.1 Pa s). Marciani *et al*. [35] investigated the effect of meal viscosity and nutrients on emptying rate in healthy subjects using MRI. The emptying rates of low-viscosity (0.06 Pa s) and high-viscosity (29.5 Pa s) meals were 6.1 and 4.7 ml min^−1^ for non-nutrient meals, respectively, and 4.1 and 3.3 ml min^−1^ for nutrient meals, respectively. Our results correspond well to these values.

When the pylorus was unable to close, our model demonstrated that the emptying rate increased to 10–30 ml min^−1^, and instantaneous retrograde flow from the duodenum to the antrum occurred during antral relaxation. This situation may be related clinically to pyloromyotomy, pyloroplasty or pyloric botox procedures, which are performed for selected indications including post-surgical gastric drainage, strictures and gastroparesis. Surgical or endoscopic opening of the pylorus may result in dumping syndrome, in which the gastric contents are rapidly emptied [36]. A pylorus that remains open can also lead to bile reflux, whereby bile flows backward from the duodenum to the antrum. Bile is an irritant to the stomach, and, if prolonged, may induce intestinal metaplasia and potentially progress to gastric cancer [37]. We showed that, when the pylorus was open during antral relaxation, retrograde flow from the duodenum to the antrum occurred as a result of the negative pressure gradient generated by recoil of the antral wall. This novel finding may offer a simple mechanism for promoting bile reflux. Notably, the occurrence of dumping and bile reflux was not restricted to the case where the pylorus remained continuously open, as both pathophysiological events could also theoretically occur due to antropyloric discoordination. Dumping syndrome was evidenced when the pylorus opened during the terminal antral contraction phase, leading to a transient major increase in the emptying rate, and bile reflux could occur when the pylorus opened during the antral relaxation phase. Although retrograde flow has been reported even in normal subjects [12,38], the coordination between the pylorus and antrum may have been incomplete for these subjects.

A two-dimensional numerical study by Pal *et al*. [23] suggested the presence of a gastric emptying road, ‘Magenstrasse’, from the pylorus to the fundus on the lesser curvature side. The gastric contents inside Magenstrasse were emptied rapidly compared with other regions of the stomach. However, our three-dimensional model predicted a different behaviour. In the case of highly viscous contents, a ‘road-like’ region appears from the pylorus to the proximal antrum, but on the greater curvature side. In the case of low-viscosity contents, the road-like region disappears, because the gastric contents are mixed homogeneously within a few minutes. While Pal *et al*. [23] prescribed the time history of the volume of gastric contents, i.e. the emptying rate, we evaluated the gastric emptying produced by peristaltic contractions, which are known to work in combination with pressure gradients to generate gastric outflow [11]. In addition, Pal *et al*. [23] only considered a high-viscosity content (*μ* = 1.0 Pa s). Magenstrasse would appear if gastric emptying is dominated by tonic contractions, and the viscosity of gastric contents is high enough.

For the present computational modelling and simulation, we assumed some idealized conditions, and they are the limitations of this study. For example, we assumed hydrostatic equilibrium to ignore any hydrostatic pressure difference across the pylorus. In reality, a hydrostatic pressure difference may be present, particularity when the proximal duodenum is empty, driving flow through the pylorus. Thus, our simulation may underestimate the emptying rate. The depth of the contraction can increase for low-viscosity contents [4], and, thus, the propulsion and retropulsion for low-viscosity contents may also be underestimated. We also assumed that gastric contents did not contain any solid components. It is well known that solid particles are not emptied until their sizes become smaller than a few millimetres. If gastric contents contain solid particles, the overall emptying would be slower because of the size effect of the particles [39]. Fluid–structure interaction modelling is necessary for the simulation of emptying of solid food particles. Computational modelling of solid foods will provide detailed information on mechanical variables, such as stress distribution, which are difficult to obtain from clinical data, and will enable us to quantitatively understand solid food emptying in disease states.

However, owing to such idealized conditions, we have successfully quantified the isolated effect of peristaltic contractions on gastric emptying. In future, this advance could be a basis for further investigating the combined effects of gastrointestinal motor functions, such as tonic contractions of the stomach, and duodenal motor functions. Furthermore, while some modifications might be necessary, our computational model would be applicable to other digestive organs, such as the oesophagus and intestine. The ability to predict *in silico* pathophysiological features that are difficult to isolate experimentally is an important outcome of gastrointestinal computational models [40], as shown here by the quantification of the effect of impaired coordination between the pyloric and antral motor functions. Although numerical simulation of the digestive system is still only an emerging field, it could therefore become an effective methodology for delivering a more comprehensive understanding of gastrointestinal physiology and related digestive diseases, in order to guide new diagnostics and therapies.

## Supplementary Material

Movie S1

## Supplementary Material

Movie S2

## Supplementary Material

Movie S3

## Supplementary Material

Movie S4

## Data accessibility

This article has no additional data.

## References

[1] Ehrlein HJ, Hiesinger E 1982 Computer analysis of mechanical activity of gastroduodenal junction in unanaesthetized dogs. Q. J. Exp. Physiol. 67, 17–29. (10.1113/expphysiol.1982.sp002611) 7079448

[2] Ehrlein HJ 1988 Motility of the pyloric sphincter studied by the inductograph method in conscious dogs. Am. J. Physiol. Gastrointest. Liver Physiol. 254, G650–G657. (10.1152/ajpgi.1988.254.5.G650) 3364566

[3] Keinke O, Ehrlein HJ 1983 Effect of oleic acid on canine gastroduodenal motility, pyloric diameter and gastric emptying. Q. J. Exp. Physiol. 68, 675–686. (10.1113/expphysiol.1983.sp002757) 6647742

[4] Pröve J, Ehrlein HJ 1982 Motor function of gastric antrum and pylorus for evacuation of low and high viscosity meals in dogs. Gut 23, 150–156. (10.1136/gut.23.2.150) 7068038 PMC1419539

[5] Wulschke S, Ehrlein HJ, Tsiamitas C 1986 The control mechanisms of gastric emptying are not overridden by motor stimulants. Am. J. Physiol. Gastrointest. Liver Physiol. 251, G744–G751. (10.1152/ajpgi.1986.251.6.G744) 3491546

[6] Keinke O, Schemann M, Ehrlein HJ 1984 Mechanical factors regulating gastric emptying of viscous nutrient meals in dogs. Q. J. Exp. Physiol. 69, 781–795. (10.1113/expphysiol.1984.sp002868) 6440209

[7] Carlson HC, Code CF, Nelson RA 1966 Motor action of the canine gastroduodenal junction: a cineradiographic, pressure, and electric study. Am. J. Dig. Dis. 11, 155–172. (10.1007/BF02239239) 5904357

[8] King PM, Heading RC, Pryde A 1985 Coordinated motor activity of the human gastroduodenal region. Dig. Dis. Sci. 30, 219–224. (10.1007/BF01347887) 3971833

[9] Brown BP, Schulze-Delrieu K, Schrier JE, Abu-Yousef MM 1993 The configuration of the human gastroduodenal junction in the separate emptying of liquids and solids. Gastroenterology 105, 433–440. (10.1016/0016-5085(93)90717-Q) 8335199

[10] Hausken T, Mundt M, Samson M 2002 Low antroduodenal pressure gradients are responsible for gastric emptying of a low-caloric liquid meal in humans. Neurogastroenterol. Motil. 14, 97–105. (10.1046/j.1365-2982.2002.00307.x) 11874558

[11] Indireshkumar K et al. 2000 Relative contributions of ‘pressure pump’ and ‘peristaltic pump’ to gastric emptying. Am. J. Physiol. Gastrointest. Liver Physiol. 278, G604–G616. (10.1152/ajpgi.2000.278.4.G604) 10762615

[12] Pallotta N, Cicala M, Frandina C, Corazziari E 1998 Antro-pyloric contractile patterns and transpyloric flow after meal ingestion in humans. Am. J. Gastroenterol. 93, 2513–2522. (10.1111/j.1572-0241.1998.00598.x) 9860417

[13] Marciani L 2011 Assessment of gastrointestinal motor functions by MRI: a comprehensive review. Neurogastroenterol. Motil. 23, 399–407. (10.1111/j.1365-2982.2011.01670.x) 21276139

[14] Schulze KS 2015 The imaging and modelling of the physical processes involved in digestion and absorption. Acta Physiol. 213, 394–405. (10.1111/apha.12407) 25313872

[15] Banerjee S, Dixit S, Fox M, Pal A 2015 Validation of a rapid, semiautomatic image analysis tool for measurement of gastric accommodation and emptying by magnetic resonance imaging. Am. J. Physiol. Gastrointest. Liver Physiol. 308, G652–G663. (10.1152/ajpgi.00095.2014) 25540229 PMC4398843

[16] Treier R, Steingoetter A, Weishaupt D, Goetze O, Boesiger P, Fried M, Schwizer W 2006 Gastric motor function and emptying in the right decubitus and seated body position as assessed by magnetic resonance imaging. J. Magn. Reson. Imaging 23, 331–338. (10.1002/jmri.20507) 16463302

[17] Ajaj W, Lauenstein T, Papanikolaou N, Holtmann G, Goehde SC, Ruehm SG, Debatin JF 2004 Real-time high-resolution MRI for the assessment of gastric motility: pre- and postpharmacological stimuli. J. Magn. Reson. Imaging 19, 453–458. (10.1002/jmri.20029) 15065169

[18] Kwiatek MA et al. 2006 Quantification of distal antral contractile motility in healthy human stomach with magnetic resonance imaging. J. Magn. Reson. Imaging 24, 1101–1109. (10.1002/jmri.20738) 17031837

[19] Pal A, Indireshkumar K, Schwizer W, Abrahamsson B, Fried M, Brasseur JG. 2004 Gastric flow and mixing studied using computer simulation. Proc. R. Soc. Lond. B 271, 2587–2594. (10.1098/rspb.2004.2886) PMC1691895 15615685

[20] Ferrua MJ, Singh RP 2010 Modeling the fluid dynamics in a human stomach to gain insight of food digestion. J. Food Sci. 75, R151–R162. (10.1111/j.1750-3841.2010.01748.x) 21535567 PMC2992692

[21] Harrison SM, Cleary PW, Sinnott MD 2018 Investigating mixing and emptying for aqueous liquid content from the stomach using a coupled biomechanical-SPH model. Food Funct. 9, 3202–3219. (10.1039/C7FO01226H) 29775189

[22] Kozu H, Kobayashi I, Nakajima M, Uemura K, Saito S, Ichikawa S 2010 Analysis of flow phenomena in gastric contents induced by human gastric peristalsis using CFD. Food Biophys. 5, 330–336. (10.1007/s11483-010-9183-y) 24931649

[23] Pal A, Brasseur JG, Abrahamsson B 2007 A stomach road or ‘Magenstrasse’ for gastric emptying. J. Biomech. 40, 1202–1210. (10.1016/j.jbiomech.2006.06.006) 16934271

[24] Imai Y, Kobayashi I, Ishida S, Ishikawa T, Buist M, Yamaguchi T 2013 Antral recirculation in the stomach during gastric mixing. Am. J. Physiol. Gastrointest. Liver Physiol. 304, G536–G542. (10.1152/ajpgi.00350.2012) 23275619

[25] Berry R et al. 2016 Functional physiology of the human terminal antrum defined by high-resolution electrical mapping and computational modeling. Am. J. Physiol. Gastrointest. Liver Physiol. 311, G895–G902. (10.1152/ajpgi.00255.2016) 27659422 PMC5130547

[26] Miyagawa T, Imai Y, Ishida S, Ishikawa T 2016 Relationship between gastric motility and liquid mixing in the stomach. Am. J. Physiol. Gastrointest. Liver Physiol. 311, G1114–G1121. (10.1152/ajpgi.00346.2016) 27789458

[27] Pullan A, Cheng L, Yassi R, Buist M 2004 Modelling gastrointestinal bioelectric activity. Prog. Biophys. Mol. Biol. 85, 523–550. (10.1016/j.pbiomolbio.2004.02.003) 15142760

[28] Spitzer V, Ackerman MJ, Scherzinger AL, Whitlock D 1996 The visible human male: a technical report. J. Am. Med. Inform. Assoc. 3, 118–130. (10.1136/jamia.1996.96236280) 8653448 PMC116294

[29] O'Grady G, Du P, Cheng LK, Egbuji JU, Lammers WJEP, Windsor JA, Pullan AJ 2010 Origin and propagation of human gastric slow-wave activity defined by high-resolution mapping. Am. J. Physiol. Gastrointest. Liver Physiol. 299, G585–G592. (10.1152/ajpgi.00125.2010) 20595620 PMC2950696

[30] O'Grady G et al. 2012 Abnormal initiation and conduction of slow-wave activity in gastroparesis, defined by high-resolution electrical mapping. Gastroenterology 143, 589–598. (10.1053/j.gastro.2012.05.036) 22643349 PMC3429650

[31] d'Humières D, Ginzburg I, Krafczyk M, Lallemand P, Luo LS 2002 Multiple-relaxation-time lattice Boltzmann models in three dimensions. Phil. Trans. R. Soc. A 360, 437–451. (10.1098/rsta.2001.0955) 16214687

[32] Mei R, Shyy W, Yu D, Luo L-S 2000 Lattice Boltzmann method for 3-d flows with curved boundary. J. Comput. Phys. 161, 680–699. (10.1006/jcph.2000.6522)

[33] Körner C, Thies M, Hofmann T, Trürey N, Rüde U. 2005 Lattice Boltzmann model for free surface flow for modeling foaming. J. Stat. Phys. 121, 179–196. (10.1007/s10955-005-8879-8)

[34] Miki T, Wang X, Aoki T, Imai Y, Ishikawa T, Katase K, Yamaguchi T 2012 Patient-specific modelling of pulmonary airflow using GPU cluster for the application in medical practice. Comput. Meth. Biomech. Biomed. Eng. 15, 771–778. (10.1080/10255842.2011.560842) 21809944

[35] Marciani L, Gowland PA, Spiller RC, Manoj P, Moore RJ, Young P, Fillery-Travis AJ 2001 Effect of meal viscosity and nutrients on satiety, intragastric dilution, and emptying assessed by MRI. Am. J. Physiol. Gastrointest. Liver Physiol. 280, G1227–G1233. (10.1152/ajpgi.2001.280.6.G1227) 11352816

[36] Tack J, Arts J, Caenepeel P, De Wulf D, Bisschops R. 2009 Pathophysiology, diagnosis and management of postoperative dumping syndrome. Nat. Rev. Gastroenterol. Hepatol. 6, 583–590. (10.1038/nrgastro.2009.148) 19724252

[37] Sobala GM, O'Connor HJ, Dewar EP, King RF, Axon AT, Dixon MF 1992 Bile reflux and intestinal metaplasia in gastric mucosa. J. Clin. Pathol. 46, 235–240. (10.1136/jcp.46.3.235) PMC501177 8463417

[38] King PM, Adam RD, Pryde A, McDicken WN, Heading RC 1984 Relationships of human antroduodenal motility and transpyloric fluid movement: non-invasive observations with real-time ultrasound. Gut 25, 1384–1391. (10.1136/gut.25.12.1384) 6392035 PMC1420190

[39] Camilleri M, Malagelada J-R, Brown ML, Becker G, Zinsmeister AR 1985 Relation between antral motility and gastric emptying of solids and liquids in humans. Am. J. Physiol. Gastrointest. Liver Physiol. 249, G580–G585. (10.1152/ajpgi.1985.249.5.G580) 4061646

[40] Cheng LK, Du P, O'Grady G 2013 Mapping and modeling gastrointestinal bioelectricity: from engineering bench to bedside. Physiology 28, 310–317. (10.1152/physiol.00022.2013) 23997190 PMC3768093

